# Unpacking the ‘process of sustaining’—identifying threats to sustainability and the strategies used to address them: a longitudinal multiple case study

**DOI:** 10.1186/s43058-023-00445-z

**Published:** 2023-06-19

**Authors:** Laura Lennox, Grazia Antonacci, Matthew Harris, Julie Reed

**Affiliations:** 1 National Institute of Health Research (NIHR) Applied Research Collaboration (ARC) for Northwest London, 369 Fulham Road, London, SW10 9NH UK; 2 Department of Primary Care and Public Health, Imperial College London, Charing Cross Campus, The Reynolds Building, St Dunstan’s Road, London, W6 8RP UK; 3 Business School, Centre for Health Economics and Policy Innovation (CHEPI), Imperial College London, South Kensington Campus, Exhibition Rd, London, SW7 2AZ UK; 4 Julie Reed Consultancy Ltd, 27 Molasses House, London, UK

**Keywords:** Sustainability, Improvement, Healthcare improvement, Quality improvement, Strategies

## Abstract

**Background:**

Although sustainability remains a recognised challenge for Quality Improvement (QI) initiatives, most available research continues to investigate sustainability at the end of implementation. As a result, the learning and continuous adjustments that shape sustainability outcomes are lost. With little understanding of the actions and processes that influence sustainability within QI initiatives, there is limited practical guidance and direction on how to enhance the sustainability of QI initiatives. This study aims to unpack the ‘process of sustaining’, by exploring threats to sustainability encountered throughout the implementation of QI Initiatives and identifying strategies used by QI teams to address these threats over time.

**Methods:**

A longitudinal multiple case study design was employed to follow 4 QI initiatives over a 3-year period. A standardised sustainability tool was used quarterly to collect perceptions of sustainability threats and actions throughout implementation. Interviews (*n*=38), observations (32.5 h), documentary analysis, and a focus group (*n*=10) were conducted to enable a greater understanding of how the process of sustaining is supported in practice. Data were analysed using the Consolidated Framework for Sustainability (CFS) to conduct thematic analysis.

**Results:**

Analysis identified five common threats to sustainability: *workforce stability, improvement timelines, organisational priorities, capacity for improvement, and stakeholder support*. Each of these threats impacted multiple sustainability constructs demonstrating the complexity of the issues encountered. In response to threats, 12 strategies to support the process of sustaining were identified under three themes: *engagement* (five strategies that promoted the development of relationships), *integration* (three strategies that supported initiatives to become embedded within local systems), and *adaptation* (four strategies that enhanced understanding of, and response to, emergent conditions and contextual needs).

**Conclusions:**

Sustaining improvements from QI initiatives requires continuous investment in relationships, resilience to integrate improvements in local systems, and flexibility to understand emergent conditions. Findings provide practitioners, funders, and researchers with a better understanding of, and preparation for, the threats associated with sustaining improvements from QI initiatives and offer insight into specific actions that can be taken to mitigate these risks. This learning can be used to inform future initiative design and support, to optimise the sustainability of healthcare improvements.

**Trial registration:**

Not applicable

**Supplementary Information:**

The online version contains supplementary material available at 10.1186/s43058-023-00445-z.

Contributions to the literature

- This paper describes how the ‘process of sustaining’ is supported in practice.
- It unpacks the process of sustaining, by describing five common sustainability threats encountered throughout the implementation of four QI initiative case studies and identifies 12 strategies used to address these threats.
- This learning provides future improvement teams with specific actions to test, address issues, and support the continuation of improved practices and outcomes.
- Findings demonstrate the need to move beyond reporting the impact of individual sustainability constructs to recognise the dynamic nature between constructs to account for the complex experiences of QI teams.

## Introduction

The number of quality improvement (QI) initiatives is increasing as healthcare organisations attempt to enhance services and care pathways to improve the quality and effectiveness of care [1–5]. QI initiatives have been established as a valuable mechanism for delivering evidence-based practice, demonstrating considerable benefits for healthcare services [3–5] including improving clinical outcomes [6–8] and increasing patient and provider satisfaction [9–12]. However, while studies have shown these initiatives can result in improvements in care, many have questioned whether they are able to maintain positive results [5, 13–15]. Lack of sustainability poses a significant risk to individuals, healthcare systems, and the wider environment and this ‘improvement loss’ can have significant consequences for patients, staff, and healthcare organisations [13, 14, 16–19]. Failure to sustain wastes limited resources, including financial investments as well as the time and effort dedicated by healthcare staff [3, 4, 18, 20, 21]. It has also been shown to negatively impact future QI initiatives as staff and other stakeholders lose enthusiasm for engaging in future programmes [22, 23]. Additionally, it has been raised as an ethical dilemma, with the social responsibility to use resources wisely and reduce waste seen as a priority for all researchers [24].

Several studies and systematic reviews have documented challenges in sustaining positive outcomes following improvement initiatives [5, 13, 17, 18, 25–28]. For example, Stirman and colleagues conducted a systematic review of 125 studies of improvements made in healthcare and found that only 45% continued delivery of programme components [16]. Conversely, some have demonstrated that sustainability can be achieved [13, 29–32]. For example, implementation of a surgical checklist found sustained reductions in 30-day surgical complications 2 years after implementation [31] and another on reducing central line-associated bloodstream infections not only sustained 10 years after initiation but also spread throughout the hospital [32]. With much of the available research focusing on reporting the success or failure to sustain, there has been little work to understand the actions and processes which lead to these diverse results [33].

### The process of sustaining

Sustainability has traditionally been viewed as an outcome to be reached at the end of implementation (e.g. the service, initiative, or activity is sustained) [34–36]. However, studying sustainability at the end of initial implementation phases fails to capture “the recursive or reflexive character of sustainability” as it does not take into account the learning and continuous adjustments that shape sustainability outcomes [34, 35]. It is also recognised that sustainability challenges occur throughout QI initiative planning, implementation, and follow-up [37, 38], leading many to acknowledge that in order to achieve sustainable improvement, sustainability planning must be considered throughout the early stages of the initiative implementation [35, 39, 40]. This has promoted a second perspective which views sustainability as an ongoing dynamic *process* operating concurrently with implementation [35, 41]. This perspective highlights the role of QI teams in responding and adapting to emerging needs to promote the continuation of improved practices, benefits, or outcomes [42]. The importance of decisions and actions taken during initiative planning, as well as support during all implementation stages, are recognised [39]. This perspective has gained popularity with implementation researchers and practitioners as it suggests that sustainability is influenced by individuals throughout initiative implementation by allowing for continuing development and adaptation in response to the needs of the system [35, 43–46].

While the ‘process of sustaining’ is increasingly discussed and understood as an accepted perspective of sustainability, there is no common description of what it entails. However, based on previous definitions [39, 46], it can broadly be defined as: *the process by which individuals and teams plan for, and act, to embed initiatives and enhance continuation of improved outcomes and practices. This includes any strategies or actions used to influence sustainability (before, during, and after implementation) which enhance prospects of continued initiative delivery and improvement.*

With very few studies taking prospective approaches to studying this process in practice [38, 47, 48], we know ‘less than we should about the mechanisms involved in adaptation and sustainability over time’ [48]. Specifically, we know very little about how individuals and teams respond and reorganise following changes and challenges to influence sustainability [47, 49, 50]. Improved description of how the process of sustaining is navigated by QI teams will provide much-needed insight into how sustainability is influenced in practice [51]. This insight will provide practitioners, funders, and researchers with a better understanding of, and preparation for, the threats associated with sustaining improvements from QI initiatives [49]. In addition, providing insight into the specific strategies used during this process is key to understanding how future initiatives can be designed and supported to optimise long-term success in future initiatives [23, 50–53].

### Aim and research questions

This paper aims to understand how QI initiatives are sustained in practice. The process of sustaining is the main area of interest for this work; therefore, the focus is not on a binary outcome of sustainment (sustained vs not sustained). Rather, we explore the threats and strategies which shape the process of sustaining. This work explores this process by investigating the threats to sustainability encountered throughout the implementation of four QI case studies and identifying how these threats are addressed through specific strategies. The following research questions will be investigated:

1. *Are common threats to the process of sustaining identified across the cases? If so, what are they?*
2. *What actions and strategies are used by QI teams to address threats to sustainability?*

## Methods

### Design

Much of the sustainability research to date has been retrospective [54]. Therefore, a prospective approach to capture real-time threats and associated responses within improvement initiatives was taken in this study. A longitudinal multiple case study design was employed to study four QI initiatives implementing evidence-based practices over a 3-year period (September 2015–September 2018). The investigation of sustainability throughout implementation aimed to make the process of sustaining (including any decision-making, actions, adaptations, and learning) explicit.

### Conceptual framework

To address the challenge associated with studying, measuring, and analysing sustainability, many have conceptualised sustainability as multiple interacting factors or constructs [21, 22, 55, 56]. Breaking the concept down into ‘manageable’ constructs is suggested to aid researchers and practitioners in navigating this complex topic [13, 21, 57]. In order to assess the individual constructs for sustainability, sustainability approaches such as frameworks, models, and tools have been developed [39, 58]. The Consolidated Framework for Sustainability (CFS) provided the conceptual basis for sustainability in this study. The CFS consolidates constructs and learning from across 62 published sustainability approaches in healthcare settings [59]. It provides a mechanism to analyse and organise sustainability data by highlighting six domains with 40 constructs that influence sustainability (Table 1).

**Table 1:** Consolidated sustainability framework. adapted from [59]

Domain	Construct
The External Environment	Awareness and raising the profile
	Socioeconomic and political considerations
	Spread to other organisations
	Urgency
Negotiating Initiative processes	Accountability of roles and responsibilities
	Belief in the initiative
	Complexity
	Defining Aims and Shared Vision
	Incentives
	Job requirements
	Workload
Resources	Resources_ General
	Funding
	Infrastructure
	Resource_Staff
	Resource_Time
The Initiative Design and Delivery	Demonstrating effectiveness
	Evidence base for the initiative
	Expertise
	Improvement Methods
	Monitoring progress over time
	Project duration
	Project type
	The Problem
	Training and Capacity Building
The Organisational Setting	Integration with Existing Programs and Policies
	Intervention Adaptation and Receptivity
	Opposition
	Organisational Readiness and Capacity
	Organisational Values and Culture
	Support Available
The People Involved	Leadership and Champions
	Ownership
	Power
	Relationships and collaboration and networks
	Satisfaction
	Stakeholder participation
	Community participation
	Patient involvement
	Staff involvement

### Setting

This study was hosted by the NIHR CLAHRC for Northwest London (CLAHRC NWL), an 11-year funded programme supporting frontline care teams to implement evidence-based practice (2008–2019). The program supported QI initiatives for a period of 18–24 months with the aim to have any improvements sustained beyond the period of support [60–62].

### Cases

The use of case studies was selected to enable the process of sustaining within initiatives to be observed [34, 63, 64]. Selecting cases from the same programme (CLARHC NWL) allowed for ‘literal replication’ in cases to uncover patterns of shared threats and strategies [65]. The four selected cases cover a range of clinical conditions and settings [66–71] (Table 2). All case interventions came from established evidence, which demonstrated improvements in patient care and/or outcomes [72–75]. Within this study, we do not seek to report on the sustainability outcomes or sustainment of the initiatives; however, all cases demonstrated continuation of specific aspects of their initiatives at 1 year post-funding (Table 2). Individual cases have reported detailed sustainability outcomes elsewhere [73, 74].

**Table 2:** Background information for the QI initiative case studies

Overview Case Studies				
Setting				
	Wellbeing [70, 71, 76]	Allergy [68]	Heart Failure [66]	MedRev [48, 49]
Initiative aim	To improve the physical health of people with severe mental illness that are admitted to a specific acute admissions ward	To improve delivery of specialist allergy services in a secondary care setting	To improve the health, quality of life, and experience of care for patients with acute heart failure	To undertake a structured medication review for patients ≥70 years who were potentially on inappropriate medicines
Intervention(s)	Physical health assessment form, training for staff on assessment and interventions, patient-held health record	Diagnosis and treatment training sessions; nurse-led asthma clinics, allergy network meetings, updated referral pathways	Admissions HF Care Bundle	Multidisciplinary team meetings, medication review tool, education and training of undergraduate pharmacists and junior doctors
Organisational setting	Acute Hospital	Site A- Integrated Care Trust Site B- Acute hospital	Acute hospital	Acute hospital
Resource	Funded by a grant from a charitable organisation.	Site A - commissioned service. Site B - funded by the Commissioning for Quality and Innovation (CQUIN) payment framework to reduce asthma admissions.	Funded by CLARHC NWL, staff time and resources were match funded by the host organisation.	Funded by CLARHC NWL, staff time and resources were match funded by the host organisation. Roll-out sites received additional funding for pharmacy time and participation.
Evidence of sustaining 1 year post-funding	Yes (Sustained adherence and delivery of physical health assessment. Integration of health assessment form on hospital’s IT system)	Yes (Maintenance of allergy proforma & child allergy booklet. Multidisciplinary team meetings continue. Adaptation including updating and adapting the review proforma)	Yes (Ongoing processes for bundle distribution and measurement. Sustained % of echocardiograms and specialist input within the recommended timeframes)	Yes (Medication reviews continued in multidisciplinary teams and mediation review tool continuing to be used)
Quality Improvement (QI) Support	CLAHRC NWL provided QI expertise, staff training, team coaching and facilitation, and evaluation support			
Timeframe	All initiatives were funded from September 2015 to March 2017 with the follow-up period extending from April 2018 to September 2018.			

### Data collection

#### Long term success tool

While the CFS provided the basis for sustainability conceptualisation and analysis, a structured sustainability planning tool, the Long Term Success Tool (LTST) [77], was used to collect data on sustainability factors from the QI team members (Supplemental file 1_LTST). The LTST was one of the 62 frameworks reviewed and integrated into the CFS and therefore there is alignment across both approach constructs and factors.

**Fig. 1:**
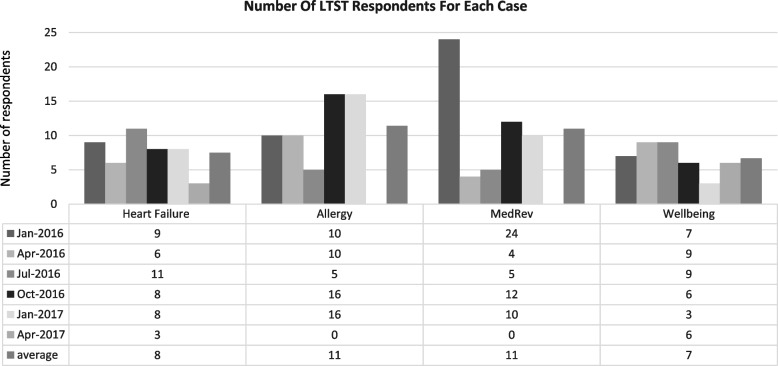
Graph displays the use of the Long Term Success Tool over time as well as the number of respondents per use across the case study teams throughout the study duration

The LTST was chosen as it provides a practical and user-friendly mechanism to collect standardised sustainability data from across the cases [78]. It is a prospective tool which investigates sustainability concurrently with implementation. This lens explicitly allows for the threats, facilitators, learning, and adaptations that influence the sustainability process to be made visible [75]. The LTST assesses 12 factors known to influence sustainability: “Commitment to the improvement, Involvement, Skills and capabilities, Leadership, Team functioning, Resources in place, Evidence of benefits, Progress monitored for feedback and learning, Robust and adaptable processes, Alignment with organisational culture and priorities, Support for improvement, and Alignment with external political and financial environment” [77]. Within the LTST questionnaire, QI team members rate factors individually using a 5-point Likert scale and can provide comments to explain ratings, highlight specific threats related to each factor, and/or suggest strategies to mitigate these risks. Team responses are aggregated to produce LTST reports (visual charts as well as comment lists for each factor) demonstrating how the initiative is performing against the given factors. For the four cases within this analysis, responses were collected quarterly throughout the funded period of each case using CLAHRC NWL online QI reporting system [79]. The LTST was used five to six times by all cases with an average of nine respondents for each case at each data collection point (Fig. 1).

#### Observation

Non-participant observation of each case took place at facilitated workshops and routine meetings (*n*= 32.5 h) to investigate if teams identified threats to sustainability and if any actions were taken (Supplemental file 2_Table 1 Observation log). Observations were recorded in a field notebook and specific meetings were audio recorded (e.g. review meetings).

#### Documentary analysis

Documentary analysis examined initiative materials, e.g. meeting minutes, presentations, review reports (*n*=65 documents, Supplemental File 2_Table 2 Documents) to investigate initiative progress, and sustainability threats and strategies.

#### Semi-structured interviews

Two rounds of key informant interviews were conducted by authors (LL and GA) to gain insight into the process of sustaining and triangulating data from observations and document analysis. The first round took place at the end of the 18-month funding period (*n*=24) and explored perceptions related to threats to sustainability and strategies proposed and employed by the teams. The second-round revisited participants (*n*=14) approximately 1 year later to explore the evolution of the threats and impact of the strategies. An interview guide was used for all interviews (Supplemental file 3_Interview Guides). A purposive sampling strategy was used to recruit interviewees from across cases (Supplemental File 2_ Table 3 Interview and focus group participant list) [80, 81]. Participants were selected based on their role within the improvement projects and their level of knowledge and specific expertise related to the initiative [81]. One case, MedRev, participated in a focus group in place of the second-round individual interviews at the request of the clinical lead and project manager. The focus group was attended by ten team members with one moderator and followed the same questions used in the individual interviews. Observational notes were taken during the focus group. All interviews and the focus group were audio recorded and professionally transcribed.

#### Data analysis

LTST scores for all cases were extracted from the online QI system into an Excel database where the Likert ratings were normalised in a numeric scale (5=Very Good to 1=Very poor). Team scores were aggregated with an overall Long Term Success Score calculated for each initiative quarterly throughout implementation. LTST data was used to understand where to focus exploration and inquiries in the qualitative data. Qualitative comments made within the LTST were uploaded to NVivo with other qualitative data for analysis of the full dataset.

A qualitative database was developed using NVivo 10 to conduct a thematic analysis of interview transcripts, documents, and observation fieldnotes [82–84]. The first stage of analysis was familiarisation which involved reading each source and revisiting, modifying, and correcting material as necessary [80–82]. A preliminary coding structure was then deductively developed using the CFS [59]. The CFS constructs provided the foundation for describing how threats impacted specific sustainability constructs. Inductive codes on strategies to address threats to sustainability were then derived, linking the strategies with overarching CFS constructs and domains. Following analysis of both the LTST scores and the qualitative data, individual case reports were drafted for each of the four case studies. The individual case reports and NVivo codes were then used to construct coding ‘word tables’ and matrices to highlight the recurrence of threats to sustainability and strategies from across the cases [65]. This format facilitated the development of cross-case analysis and conclusions [65]. The data were then summarised into narratives with quotations to highlight sustainability threats and strategies.

## Results

Results are presented in two sections. First, common threats to the process of sustaining are identified and described from across the cases. Second, shared strategies taken to address threats and mitigate risks to support sustainability are discussed.

### Identifying threats to the process of sustaining

Each case experienced multifaceted issues undermining the process of sustaining within the initiatives, with the timing, frequency, and impact varying across cases. The analysis identified five common threats, each impacting multiple sustainability constructs, demonstrating the complexity of the issues encountered (Table 3).

i. *Workforce stability*: All cases experienced turnover of staff, particularly nurses and junior doctors. This turnover created issues in handover and continuity of initiatives as staff moved on. It impacted the ability of initiative rationale and measurement to be communicated and carried out adequately and consistently. Ultimately, this compromised initiative memory as significant experience and expertise was lost.
ii. *Improvement timelines*: Producing evidence of benefits within the funded improvement project timeline was a shared threat across case studies. This was largely due to initiative planning and set-up taking longer than expected, limiting the amount of time the teams had to collect measures and perform meaningful evaluation of the initiative. With limited evidence of how the initiatives were producing improved outcomes, initiatives struggled to gain continued support and further buy-in from their organisations.
iii. *Competing organisational priorities*: Inconsistent support for improvement initiatives from organisational leaders was a recognised threat to sustainability. Participants described competing priorities such as fluctuating organisational strategies, changes to infrastructure and systems, financial cuts, and emerging innovations. This created opposition, hindering initiatives’ ability to garner support and gain necessary resources or integrate changes within organisational systems.
iv. *Capacity for improvement*: Improvement initiative work was often conducted on top of healthcare staff ‘day jobs’ and therefore relied on core individuals or groups. This was a significant threat to sustainability because without these individuals both delivery and data collection of the initiative was compromised.
v. *Maintaining stakeholder support*: Teams struggled to garner and maintain stakeholder (staff and service users) support and engagement throughout the initiatives. Without involvement, teams had limited ability to understand the experience of staff and patients or the need for adaptation to tailor improvements to preferences and needs. Additionally, without specific involvement from service users or patients, team members felt that the initiatives would not have the necessary backing and ‘real life’ impact stories to promote initiative continuation.

**Table 3:** Sustainability threats encountered across the cases and their impact on CFS sustainability constructs

**Sustainability challenge**	**CFS Construct:** description of threat impact on construct	**Representative quotation**
**1. Workforce stability:** caused issues in handover and continuity as staff moved on and new staff were brought in.	**Demonstrating effectiveness:** unstable workforce compromised the ability of initiative rationale, learning, and measures to be communicated adequately and consistently.	“You have turnover of junior doctors every six weeks…so, again, just when you've got them comfortable and happy to use it then they change into another. So it's always going to be a challenge.” (I21_QI manager)
	**Training and capacity building:** Staff turnover required extra training as well as continual engagement and establishment of working relationships with recruits and partners.	“You need to think about new training awareness…that’s a constant challenge actually because your staff group is always changing.” (I2_Clinical lead)
	**Organisational readiness and capacity:** Staff turnover compromised initiative delivery and organisational memory as staff left.	“I can point to at least five people if not more within the original project team, that have got promoted during that time….you’ve taken away a lot of that ‘know how’ that was generated.” (I6_QI manager)
**2. Improvement timelines:** limited improvement project funding and timelines	**Evidence base for the initiative:** limited timeframes compromised the ability to perform meaningful evaluation of the improvement work during and at the end of the initiative.	“We’ve had some good things happening with the project…but I think it’s the frustration that things haven’t come on as well or as fast maybe, as we would have thought.” (I3_Service User)
		“A lot of people feel that even with them working quite hard there is still not the evidence they want to see. There’s still not the patient impact that they want to see.” (Allery_12 month review transcripts)
	**Belief in the initiative**: limited QI timelines compromised the capacity to demonstrate impact to staff and funders by hindering the generation of the evidence base needed to sustain the initiative.	“Outcome measures (A&E attendances) look positive, but reliability and (project) attribution are unclear. Process measures (management plan, skin prick testing etc.) look promising, but need to be looked at in more detail.” (LTST Questionnaire response_Allergy _February 2016)
**3. Competing organisational priorities:** difficulty operating within organisational limitations and shifting strategies as well as remaining relevant to organisational leaders.	**Resources**: Shifting strategies compromised the initiatives’ ability to garner support and buy-in as well as the capacity to gain access to necessary resources to continue the initiatives.	“Projects have clearly got over it by committed people within the organisation just going, hell, we’re going to do it, and doing it….you’ve done it regardless of the fact that actually the funding and administration support has not been offered.” (Allergy_18 month review transcript)
	**Integration with existing programs and policies:** organisational limitations compromised ability of teams to embed changes within existing programs, processes, and policies.	“In a big organisation like this there’s every chance that someone else will decide there’s some other way of doing things and then all of a sudden this all goes out the window…and things change so much in the NHS (National Health Service) that good things just get wiped out sometimes.” (I1_Clinical lead)
	**Opposition**: competing priorities compromised staff motivation as well as local and organisational support.	“There seems to be minimal organisational support within the project; sometimes I felt that we (core team) have been working against a whole organisation to improve children's health outcomes.” ( LTST Questionnaire response_Allergy_February 2016)
**4. Capacity for improvement:** difficulty to conduct improvement work without having dedicated time and therefore relying on core individuals.	**Team functioning:** limited staff capacity compromised the fair distribution of workload and responsibilities.	“In the short term you’re delivering the improvements in the project, but in the longer term you’re going to have a detrimental effect on the sustainability of the project…if it’s somebody’s job to do it now, then what happens when they’re not there or what happens when they leave.” (I6, QI manager)
	**Job requirements:** improvement work increased workload and responsibilities for staff with no additional resources.	“We definitely will need admin support…I can do that now…because I’m only covering two specific areas...I don’t think they realised how much admin support would be needed.” (I19_Nurse)
	**Ownership:** Lack of ownership as projects are seen as personal projects rather than organisational priorities, compromising the organisational ownership of the initiative.	“The project seems to belong to somebody rather than to the organisation and I think that's been the biggest factor that I’ve seen in sustainability, is the lack of organisational ownership of projects, they think it belongs to an individual consultant or nurse or even a team of people, not the organisation.” (I6_QI manager)
**5. Stakeholder support:** difficulty to garner and maintain support and stakeholder engagement in the initiatives.	**Intervention adaptation and receptivity:** Lack of stakeholder engagement compromised the ability to understand the experience of staff and patients and how to tailor improvements to their preferences and needs.	“If what we’re doing is clunky and alienating and unmanageable for patients, then they won’t go along with it either, so I think having them, gave us a bit of a check and a balance with that to make sure that we were doing something that was likely to be acceptable to patients and service users.” (I2_Clinical lead)
	**Staff involvement and community participation:** Lack of support from stakeholders resulted in missed opportunities for raising awareness, championing, and influencing decision-making.	“True involvement, engagement of patients, I would say that this is a weakness of the project and…it’s very hard to know whether that would have created some pressure with the hospital management or the trust management, but it may have been a positive influence on the involvement of GPs.” (I20_QI manager).

### Strategies to address threats to sustainability

To respond to threats, the cases undertook a number of actions to address issues and mitigate risks. Twelve strategies to address threats to sustainability were identified from across the cases and grouped into 3 emergent themes: *engagement, integration, adaptation* (Table 4). Strategies are not reported as linear or direct responses to specific threats as findings demonstrated that teams used varying combinations of strategies to address threats dependent on their settings, priorities, available resources, and ability to act within specific domains. Therefore, each strategy had a wide-ranging impact and supported teams to manage multiple interdependent challenges.

**Table 4:** Summary of strategies to address threats to sustainability employed across the case studies

**Theme**	**Strategy**	**Example actions undertaken**	**Representative quotations**	**Initiative Use (yes/no)**			
				**Wellbeing**	**Allergy**	**Heart Failure**	**MedRev**
**Engagement**	**1. Engaging with senior leaders** to gain support and buy-in.	• Teams used governance or trust meetings as opportunities to communicate the rationale and impact of the work and to foster belief in the importance of the initiative.	“Every clinical governance meeting we bring an update on what has happened and we use it to continually drive the bundle…We just need to give them something to help keep them motivated” (I22, Service manager)	Y	Y	Y	Y
	**2. Involving patients** to act as a catalyst for change	• Teams involved service users in coproducing initiative material and products to facilitate usability and effectiveness. • Patients partnered to spread awareness and champion the initiative in other settings. • Teams utilised patients and their experience to inspire action to continue the improvement work.	“There is something I can do and I will…bang my fist on the table to explain (MedRev)…in case there is someone at (another hospital) who hasn’t heard about it.” (I8_Patient representative)	Y	N	N	Y
	**3. Building collaboration and networks** to foster multi-disciplinary approaches, cross-site learning, new relationships, and future collaborative working.	• Teams established networks and collaborations by attending forums, organising collaborative funding applications, setting up networks or multidisciplinary team meetings.	“Having this working relationship across all five sites in North West London that’s not going to go away and now there are other projects that we’ve...actually put in bids together and that seems like a really natural thing to do.” (I7_Project Manager)	N	Y	Y	Y
	**4. Building in and planning for accountability and ownership** of the initiative to allow staff to better understand and balance workload and responsibilities.	• Teams built accountability for the work into workforce planning, clearly defined job roles and allocated tasks.	“We’ve built that into some of the job plans now…that’s part of the role so one of the benefits of people leaving and…getting other people in when they start you go that’s just what you do.” (I1_Clinical lead)	Y	N	N	Y
	**5. Ongoing promotion of the initiative** to maintain momentum, to further promote interest and engagement, and to heighten staff morale and belief in the initiative.	• Teams promoted initiatives through forums, publications, meetings, newsletters, award nominations, conferences, and email updates.	“I did all the data collection weekly sending (it) out like hey, this is a really good example and encouraging people to get together and talk through examples of cases.” (I7_Project manager)	Y	Y	Y	Y
**Integration**	**6. Consistent and continuous training and capacity building** to enable a wider workforce to deliver the initiative and ensure staff have the capacity to consistently deliver the work despite turnover.	• Teams built capacity by adding information on initiatives to induction presentations and packages and having consistent education sessions.	“Training one person or having a consultant doing outreach long term is not the answer. We have to build the education model in to it.” (Allergy_6 month review transcripts)	N	Y	Y	Y
	**7. Embedding measurement and monitoring** to enable feedback to staff and stakeholders to encourage continuation of the work.	• Teams displayed progress using graphs and charts on the ward to celebrate continuation of data collection over time. • Teams fed-back process measures to show incremental changes to services to provide information to funders and leaders to support continuation of the work.	“It’s really good to keep looking at what we did…and keep measuring, because we’re still seeing improvements and all the time you’re still seeing improvements you want to keep on measuring, because it’s good.” (I14_Clinical lead)	Y	Y	Y	N
	**8. Integration of changes in systems, processes, and funding mechanisms** to support initiative staffing, infrastructure and spread.	• Teams integrated documents onto online IT system and linked initiatives with existing funding mechanisms	“So we’ve got now a process by which…they’ve almost bought in forever, because we’re saying, well if you take this away, you’re not going to get your best practice tariff.” (I25_Project manager)	Y	Y	Y	Y
**Adaptation**	**9. Identifying and applying for further funding** to continue and spread the services	• Teams identified and applied for further funding such as CQUIN targets, and fellowship grants and prepared business cases for commissioners.	‘My colleague has applied for funding…the natural continuation is actually get the community to get engaged with the project itself.” (I11_Pharmacist)	Y	Y	Y	Y
	**10. Expanding the initiative** to reach more patients, enhance equity of services and garner further impact and support from staff and organizational leaders	• Teams rolled out initiatives to further wards and expanded the programme to community settings.	“The idea is to roll out across the unit and into the community, and I think if this becomes the embedded way of working across the unit and the community, it’s got a far better chance of success.” (I6_QI manager)	Y	Y	Y	Y
	**11. Reducing the scope of the initiative** to ensure feasible delivery and to build in mechanisms for continuation by 'starting small' and understanding how best to deliver the initiative in practice.	• Teams reduced their project target or postponed spread of the initiative to other sites.	“Taking our time and really looking into each aspect of the project has helped us sustain it…if we went into it, to a project the size it is now, I don’t think we would’ve ever been able to sustain that…if you start small and you get that right and you know what works, what doesn’t, you can then scale it up to a bigger project based on that.” (I19, Nurse)	Y	Y	N	N
	**12. Adaptation of the initiative processes and products** to allow each improvement to be tailored to the setting and existing limitations, staff preferences, and emerging evidence.	• Teams adapted interventions by iteratively changing documents and responding to staff feedback on how forms or processes were working.	“Every three, five, ten years the guidelines change slightly, so we constantly have a group of specialised people that look at it…and make sure that we’re actually on the right page.” (I22_Service manager).	Y	Y	Y	Y

#### Engagement

Five strategies promoted the recognition, use, or development of relationships, partnerships, and connections within systems to support the process of sustaining.

##### 1. Engaging with senior leaders

All cases attempted to engage and gain buy-in from senior leaders within their settings. This was key to sustaining due to leaders’ ability to advocate for the initiative and gain further commitment from staff members. Teams worked strategically to identify and target leaders across their organisations to foster belief in the importance of their initiatives. For example, the Heart Failure team gained access to leaders at quarterly governance meetings where they prepared presentations to communicate how the initiative could support organisational priorities. Actions such as these enabled the teams to maintain support for the work and gain ongoing commitment from staff.

##### 2. Involving patients

Two cases used the strategy of forming relationships with, and involving, service users in their initiatives. This aided the process of sustaining in multiple ways. The first was the ability of patients to act as a catalyst for change and a ‘pull’ for the improvement work. Their capacity to push the team and inspire action was felt as a key stimulus to continuing the improvement work. The second was the role of patients in informing key initiative outputs. For example, in Wellbeing, service users led the design of a coproduced patient-held health record which enhanced its usability and effectiveness for patients in the future [71]. The third benefit of involving patients was related to their ability to maintain momentum for the work by spreading awareness and championing the initiative in other settings. For example, a patient representative in MedRev campaigned for the work at multiple hospital Trusts.

##### 3. Building collaboration and networks

Inter-professional collaboration between staff groups was important to the process of sustaining as it supported staff to engage in multidisciplinary approaches to deliver initiatives effectively. This enabled teams to build lasting relationships to maintain the work in the future. Networks and collaborations were established in different ways. Some teams set up network meetings and attended forums to build contacts, while others organised collaborative funding applications or began multidisciplinary clinical meetings. This strategy provided a platform for continual engagement with staff as well as an opportunity to meet new stakeholders to gain ongoing support for the initiatives.

##### 4. Planning for accountability and ownership

Participants highlighted the importance of explicitly outlining workload and responsibilities to ensure staff were aware of their role in QI initiatives. This strategy maintained continued delivery of the initiative and allowed staff to share responsibilities so that the workload would not be reliant on individuals. This involved teams informing workforce planning and adapting job roles and descriptions to allocate tasks and ensure responsibilities were clear. For example, in MedRev, accountability for the initiative was built into job descriptions by assigning staff-specific roles in medication review.

##### 5. Maintaining momentum through ongoing promotion

All cases engaged in the ongoing promotion of the initiative, raising awareness, and highlighting the evidence base surrounding each intervention. This was done through presentations at clinical forums, multi-disciplinary team meetings, and conferences, as well as within publications, newsletters, and email updates. This strategy promoted further interest and engagement and acted as a reminder to staff to continue delivery. It also enabled staff to build morale, as the more initiatives were publicised, the more opportunity the team had to be proud and share their achievements. For example, in Wellbeing, the team reflected that when the project was picked up by organisational leaders as an example of improved practice at the hospital, delivery was reinforced, and more ownership was felt by staff.

#### Integration

Three strategies enhanced initiative integration within systems to support the process of sustaining. These strategies helped participants not only understand initiative progress but also were crucial for consistent delivery and maintenance.

##### 6. Consistent and continuous capacity building

To ensure staff had the capacity to consistently deliver the improvement work, three cases developed some form of continuous training to support the process of sustaining. This included adding initiative information to induction presentations and packages, linking the initiative with undergraduate teaching and postgraduate diplomas, and having consistent training sessions. For example, in MedRev, the addition of de-prescribing material to junior doctor and pharmacist induction training built a foundation of knowledge in early career staff. Such strategies embedded initiatives into routine practice and enabled a wider workforce to understand the need for the initiatives.

##### 7. Embedding measurement and monitoring

The ability to monitor progress and have measures in place was identified as facilitating the process of sustaining. Specifically, teams collected process measures to act as proxy measures of success before the broader impact could be ascertained. For example, participants in Allergy described how their ability to report measures, such as the number of referrals, allowed the team to demonstrate changes to pathways to interest and consistently engage healthcare commissioners. Similarly, in Wellbeing, monitoring the number of documented physical health checks completed enabled the project to show incremental changes to the service. This strategy provided essential information to funders and leaders to support continued interest in the work.

##### 8. Impacting organisational memory through system integration

All cases attempted to integrate changes within their local systems. Integration occurred across multiple levels within the cases (e.g. integration within documentation processes, monitoring systems, training, and funding mechanisms). MedRev integrated their discharge summary for medication review into an online documentation system, while Wellbeing embedded their physical health assessment form into their online IT system. This strategy supported consistent data collection and feedback. Additionally, the Heart Failure bundle was integrated into existing funding streams—the Commissioning for Quality and Innovation (CQUIN) payment framework [40] and the Best Practice Tariff (BPT) for HF patients [85]. This allowed the initiative to monitor and deliver a standard of care while receiving payment for meeting specific targets. This strategy ensured that some form of legacy of the initiative existed beyond the knowledge of individual staff members or groups.

#### Adaptation

Four strategies highlighted the importance of teams understanding emergent conditions and contextual needs to support the process of sustaining.

##### 9. Identifying and applying for further funding

To support adequate time to produce evidence of benefits, all cases were identified and applied for further resources or funding. For example, Heart Failure and Allergy prepared business cases for their initiatives which were presented to commissioners to support continued initiative staffing. MedRev and Wellbeing staff applied for fellowship grants to support initiative spread in other sites. This strategy provided initiatives with the opportunity to continue the work and maintain staffing structures. Receiving extra funding was also seen as a proxy measure of success demonstrating to staff and leaders the importance of the ongoing delivery of the work.

##### 10. Expanding the initiative to other sites and settings

All cases identified spread as a strategy to support the process of sustaining improvements. During the study period, Wellbeing rolled out to five further wards within their hospital and Allergy established two further allergy clinics in the community. Participants described two reasons spread was perceived to be valuable to sustaining improvements. First, the teams wanted to ensure the accessibility of their service and reach greater patient populations as this was anticipated to increase the potential impact and evidence for the work. Second, team members perceived that an initiative acting on a larger scale would be more likely to garner long-term support from staff and organisational leaders.

##### 11. Reducing scope of the initiative

Two cases made the decision to reduce or change the scope of their initiatives to deliver initiatives within a given capacity. In Allergy, this involved choosing to reduce their project from a broad intervention targeting all allergy illnesses to asthma services only. In Wellbeing, the team decided to postpone the spread of the initiative to community sites. The rationale for these actions was to foster lasting change which could realistically be delivered within the available time and resources. This strategy not only allowed team members to understand how to pragmatically deliver the initiative in practice, but it also gave the teams an opportunity to sufficiently consider how to build in mechanisms for continuation.

##### 12. Adaptation of the initiative processes and products

Each case worked to understand and respond to contextual needs by adapting initiatives to staff feedback, organisational limitations, and emerging evidence. For example, multiple cases described how they made iterative improvements to documents such as the patient-held health records, care pathway proformas, or care bundles. These iterations were important to the process of sustaining as they allowed each improvement to be adapted to organisational characteristics. These changes were noted as being necessary to the continued delivery of the improvements, as teams were able to develop processes and outputs that best suited their given needs.

## Discussion

This work responds to the call for health services research to identify and explain not only the outcomes of improvement, but also the influences and processes supporting these results [86, 87]. Findings build new learning by describing the process of sustaining, specifically outlining how teams address threats to sustainability during implementation and describing real-world strategies employed to support the process. This work provides unique empirical contributions to the field by consolidating this learning from across different intervention types and settings. Through cross-case analysis, we were able to observe not only what actions teams took to support the process of sustaining but also identify the potential actions which were not employed across the cases to further support the process of sustaining. This learning provides future QI teams with specific actions to test in practice to address issues and support the continuation of improved practices and outcomes.

Findings demonstrate that despite unique circumstances and diverse disease areas, initiatives were impacted by five common threats to sustainability*: workforce stability, improvement timelines, organisational priorities, capacity for improvement and stakeholder support.* To address threats and support the process of sustaining, teams engaged in active problem solving, making changes and adjustments to systems, intervention processes, and plans. This highlights the role of individuals in responding and adapting to improve initiative design and characteristics to maintain improvements in care [46].

Five strategies promoting the recognition or development of relationships within systems were identified. Improvement teams built and maintained numerous relationships, connections, and partnerships across their systems. Fostering these interdependencies is crucial to sustainability as it allows teams to share information, organise implementation and delivery, and make decisions to accomplish tasks [88]. The link between engagement and sustainability has been supported elsewhere, with the literature demonstrating that collaboration between diverse stakeholders allows for shared understanding of problems to be established and aids in the creation of responsive and effective interventions [20, 21, 89, 90]. Uniquely, this work highlighted the specific role of service users and patients in contributing to initiative sustainability. This finding provides further evidence on reports that patient participants embrace sustainability as one of their core responsibilities and use their existing networks within healthcare organisations to raise awareness [91].

Findings also proposed three strategies to increase initiative integration within systems. These strategies provide insight into how the initiatives can be built into current systems and processes to foster continuation. The value of integration in sustaining improvements has been promoted in a number studies [18, 23, 61, 92]. For example, Martin et al. described how impacting organisational memory through integration in systems influences stakeholder support and decreases the chance of staff making further changes to interventions [93].

Finally, the role of adaptation to support sustainability was highlighted within four strategies. These strategies demonstrate the importance of fostering learning, feedback, and responsiveness in improvement teams [38, 92]. Research has indicated changes to interventions are often desirable to support initiative sustainability, ‘especially if changes reflect additions to the intervention rather than subtractions from it’ [94]. However, a fundamental challenge in studying sustainability is the tension that exists between the continuation of interventions as originally designed, and the need to adapt across different settings [39, 95, 96]. While the presented strategies provide insight into the types of adaptations viewed by improvement teams as necessary to sustain improvements in practice, further research is required to study any trade-offs between the sustainability and adaptation [54].

### Strengths and limitations

The opportunity to study sustainability as a dynamic, prospective process throughout implementation was critical to gain insight into how sustainability of improvements is influenced in practice [13, 14, 16]. To our knowledge, this is the first longitudinal study to examine the process of sustaining in detail and present common sustainability strategies which have been utilised across different intervention types and settings. Although this study offers valuable insight into how QI initiatives are sustained in practice, there are key limitations which should be considered.

First, a limitation of case study research is the extent to which generalisations can be drawn from a small number of cases [97]. As our sample was relatively small, we cannot establish the probability that data is representative of other improvement initiatives [98]. Equally, as all cases operated within the same QI context, the findings may not be directly transferable to other QI programmes. However, they can provide valuable understanding of the types of threats to anticipate, and strategies to employ to support sustainability which can be considered and tested within future research.

Second, while the strategies presented in this study demonstrate how specific QI teams addressed threats to support sustainability in practice, we cannot say if these were the ‘right’ strategies to use. While evidence for several of the presented strategies has been established, others require further exploration. Specifically, further work is needed to understand the potential unintended consequences of the proposed strategies to ensure teams can make informed decisions when sustaining. For example, while strategies such as adapting initiatives or reducing scope may ensure feasible delivery, they may also result in changes to anticipated outcomes or fewer people receiving the improvement. This may mean the potential impact of the initiative is diminished. Interestingly, cases also described that spreading initiatives was a strategy for sustainability. This work has demonstrated that spreading initiatives aided teams to broaden their population base, increase potential impact and evidence of benefits, and promote legitimacy of the initiative. Although this finding provides insight into the motivation of teams to spread improvement, there is limited evidence on if, and how, spread can support or hinder sustainability [99–102].

Finally, we cannot say how each strategy directly impacted sustainment. Due to the complexity and inherent interdependency of sustainability constructs, explanations describing causal mechanisms between actions taken and impact on sustainability were not feasible and beyond the scope of this study [54, 103–105]. While we able to gather early evidence of initiatives sustaining in the analysis (Table 1), we were unable to follow them beyond this point. As sustainability is likely to be measured on a gradient with partial sustainment of specific aspects of an initiative as well as adaptations to promote continuous improvement, future researchers are encouraged to consider and report multifaceted sustainability outcomes rather than binary outcomes for sustainment [39, 106].

### Implications for research and practice

QI initiative success is often judged within strict improvement timeframes, requiring QI teams to establish unrealistic conditions to show rapid improvement (e.g. by employing more staff for the project duration). This limits the potential sustainability of these initiatives once additional funding is removed [107]. To achieve sustainable improvement, researchers, funders, and practitioners must acknowledge that embedding improvement takes time, allowing interdependent practices, systems, and infrastructure to respond and adapt to new ways of working. Funders and healthcare managers should work with practitioners to understand how they can support implementation in ‘real world’ conditions to enhance their ability to embed and sustain changes. Employing the strategies suggested within this study, early on and throughout initiative implementation, can support QI teams to build the foundations required to support long-term change and continuous improvement.

In order to sustain, teams must engage in continuous threat identification and active problem solving, making changes and adjustments to interventions, processes, and systems. Our findings demonstrate that teams need to be flexible, creative, and resilient to persist through continuous challenges and learn to adapt to meet needs. These skills have become increasingly important for future initiatives to promote sustainability in constantly changing and increasingly challenging environments [107]. With few teams explicitly taught these skills, future work should consider how to adequately prepare teams for the practical reality of sustaining improvements in healthcare [108].

The application of the CFS in reviewing sustainability constructs across the cases was a useful basis for initial data organisation, interpretation, and analysis. However, findings suggest that there is value in moving beyond reporting the impact of individual constructs to describe complex experiences as seen by improvement teams [16, 39]. Reducing complex issues to single constructs such as ‘leadership’ or ‘resources’ poses a risk, as it suggests that addressing that construct alone may resolve issues. Our results have demonstrated that sustainability threats require teams to navigate multiple interacting constructs using multiple strategies. For example, while staff turnover was a ‘resource’ issue, interacting factors like staff engagement, training, and workload also needed to be simultaneously considered in the analysis of sustainability threats. This conclusion extends previous work which found that complex phenomena, such as sustainability, require recognition of the dynamic nature within and between constructs and cannot be fully understood in isolation [54, 103–105]. Future sustainability studies are therefore encouraged to provide nuanced and representative accounts of what to expect in sustaining improvement.

## Conclusion

Sustaining improvements in healthcare settings poses a significant challenge for QI teams, healthcare planners, and staff [16, 58]. Given the lack of practical guidance and direction within the current literature, it is critical that knowledge on how to enhance the process of sustaining is shared and tested across QI programmes [5]. This paper provides insight into the process of sustaining and how it is navigated by QI teams in practice. While initiatives may have unique implementation journeys, common threats to sustainability are likely to be encountered, and specific strategies can be used to address obstacles to support sustainability.

## Supplementary Information

**Additional file 1.** Long Term Success Tool.

**Additional file 2: Table 1.** Observation log. **Table 2.** Documents. **Table 3.** Interview and focus group participant list.

**Additional file 3.** Interview Guides.

## Data Availability

The datasets used and/or analysed during the current study are available from the corresponding author on reasonable request.

## Abbreviations

CFS: The Consolidated Framework for Sustainability
CLAHRC: Collaboration for Leadership in Applied Health Research and Care
CQUIN: Commissioning for Quality and Innovation
LTST: Long Term Success Tool
NHS: National Health Service
NIHR: National Institute of Health Research
NWL: Northwest London
QI: Quality improvement
WISH: Web Improvement System for Healthcare
