# Supplementary material for: Unpacking the ‘process of sustaining’—identifying threats to sustainability and the strategies used to address them: a longitudinal multiple case study
Source: Implement Sci Commun. 2023 Jun 19;4:68. doi: 10.1186/s43058-023-00445-z (PMC10278281; doi:10.1186/s43058-023-00445-z)
Supplement: Supplementary file 1 — Additional file 1. Long Term Success Tool. [file 43058_2023_445_MOESM1_ESM.pdf]

©NIHR CLAHRC
Northwest London

Long Term Success Tool

This tool aims to aid you in planning for long term success of your work. You will be asked to rate 12 factors that have been identified to impact long term success from current literature and evidence.

Each rating should represent an overall impression of how you believe your project is doing. Please use the boxes to highlight any comments or actions needed to address the factors.

A. Please specify which project you are completing the form for:

B. Please rate the project in the following factors:

1. Commitment to the improvement

My team understands what the project is trying to achieve and believe this work will lead to improved processes and outcomes.

❍ Very Good ❍ Good ❍ Fair ❍ Poor ❍ Very Poor ❍ No Opinion ❍ Don't Know

Comments and actions:

2. Involvement

a) I have the opportunity to input into the project and I feel a sense of ownership towards the work. I am able to express my ideas freely which are openly considered by the team.

❍ Very Good ❍ Good ❍ Fair ❍ Poor ❍ Very Poor ❍ No Opinion ❍ Don't Know

Comments and actions:

b) There is wide breadth of involvement from stakeholders including patients and members of the public who regularly feed into the project.

❍ Very Good ❍ Good ❍ Fair ❍ Poor ❍ Very Poor ❍ No Opinion ❍ Don't Know

Comments and actions:

3. Skills and capabilities

Staff have the necessary skills to deliver the improvement. Training and development opportunities are available to all staff, volunteers and other people involved.

❍ Very Good ❍ Good ❍ Fair ❍ Poor ❍ Very Poor ❍ No Opinion ❍ Don't Know

Comments and actions:

4. Leadership

My project has supportive and respected leaders and/or champions who advocate for the improvement, communicate the vision, and effectively manage the process.

❍ Very Good ❍ Good ❍ Fair ❍ Poor ❍ Very Poor ❍ No Opinion ❍ Don't Know

Comments and actions:

5. Team functioning

My project team is working well together. There are clear responsibilities for individuals and the work is shared across the team and does not rely on particular individuals.

❍ Very Good ❍ Good ❍ Fair ❍ Poor ❍ Very Poor ❍ No Opinion ❍ Don't Know

Comments and actions:

Please turn over

Long Term Success Tool

6. Resources in place
My project has financial support that will allow the improvement to achieve long term success. We have the necessary staff, material and equipment. I am given sufficient time to dedicate to the improvement.

❍ Very Good ❍ Good ❍ Fair ❍ Poor ❍ Very Poor ❍ No Opinion ❍ Don't Know

Comments and actions:

7. Progress monitored for feedback and learning
There is a monitoring system in place that allows the team to collect, manage and regularly review data. Feedback from the project is shared with me and other stakeholders on a regular basis.

❍ Very Good ❍ Good ❍ Fair ❍ Poor ❍ Very Poor ❍ No Opinion ❍ Don't Know

Comments and actions:

8. Evidence of benefits
There is evidence of benefits emerging from the project and this evidence is regularly communicated and visible to staff and patients.

❍ Very Good ❍ Good ❍ Fair ❍ Poor ❍ Very Poor ❍ No Opinion ❍ Don't Know

Comments and actions:

9. Robust and adaptable processes
There is the opportunity to adapt the project to reflect local needs, setting and emerging evidence. Adaptations are documented and the successes and failures of changes are reported.

❍ Very Good ❍ Good ❍ Fair ❍ Poor ❍ Very Poor ❍ No Opinion ❍ Don't Know

Comments and actions:

10. Alignment with organisational culture and priorities
The improvement my project is trying to achieve is aligned with the strategic aims and priorities of the organisation(s) we work within and our work contributes to these aims. Our work is supported by the policies and procedures within the organisation.

❍ Very Good ❍ Good ❍ Fair ❍ Poor ❍ Very Poor ❍ No Opinion ❍ Don't Know

Comments and actions:

11. Support for improvement
There are values and beliefs in my organisation(s) that emphasise the need to improve. Staff and management are supportive of improvement initiatives and continuous improvement is a priority for the organisation, staff and patients.

❍ Very Good ❍ Good ❍ Fair ❍ Poor ❍ Very Poor ❍ No Opinion ❍ Don't Know

Comments and actions:

12. Alignment with the political and financial environment
My project exists in a supportive economic and political environment. My team is aware of external pressures and incentives that may influence the project.

❍ Very Good ❍ Good ❍ Fair ❍ Poor ❍ Very Poor ❍ No Opinion ❍ Don't Know

Comments and actions:

Thank you for your time

Individuals may reproduce or adapt this content for their use provided that proper attribution is given to the appropriate source. The recommended citation for this tool is:

Lennox L, Doyle C, Reed J, Bell D. What makes a sustainability tool valuable, practical, and useful in real world healthcare practice? A qualitative study on the development of the Long Term Success Tool in Northwest London. BMJ Open. 2017;7(e014417):1–13.
