# Supplementary material for: Unpacking the ‘process of sustaining’—identifying threats to sustainability and the strategies used to address them: a longitudinal multiple case study
Source: Implement Sci Commun. 2023 Jun 19;4:68. doi: 10.1186/s43058-023-00445-z (PMC10278281; doi:10.1186/s43058-023-00445-z)
Supplement: Supplementary file 2 — Additional file 2: Table 1. Observation log. Table 2. Documents. Table 3. Interview and focus group participant list. [file 43058_2023_445_MOESM2_ESM.pdf]

Supplementary File 1: Methods tables

Table 1: Observation Log

Programme	Date	Initiative	Meeting type	Time	Observation hours
1	27/11/2014	MedRev	Team meeting	12:00-14:00	2
1	26/01/2015	Allergy	Team meeting	9:00-12:00	3
1	12/06/2015	Wellbeing	Team Meeting	15:30-17:00	1.5
1	12/08/2015	Allergy	Team meeting	11:30-14:00	2.5
1	13/08/2015	MedRev	Team meeting	9:30-11:30	2
1	23/10/2015	Allergy	Review meeting	16:00-17:00	1
1	02/11/2015	Heart Failure	Team meeting	15:30-17:00	1.5
1	15/11/2015	Wellbeing	Team meeting	15:30-17:00	1.5
1	25/11/2015	MedRev	Programme support meeting	9:00-11:00	2
1	02/12/2015	Allergy	Review meeting	15:30-16:30	1
1	27/01/2016	Heart Failure	Programme support meeting	9:00-11:00	2
1	13/02/2016	Heart Failure	Team meeting	15:00-17:00	2
1	02/03/2016	Allergy	Review meeting	15:30-16:31	1
1	04/03/2016	Allergy	Review meeting	15:00-16:00	1
1	17/03/2016	MedRev	Team meeting	10:00-11:00	1
1	06/04/2016	Wellbeing	Programme support meeting	9:00-10:30	1.5
1	08/04/2016	Wellbeing	Team Meeting	15:30-17:00	1.5
1	20/07/2016	MedRev	Learning event	13:40-14:40	1
1	22/02/2017	Wellbeing	Programme support meeting	9:00-10:00	1
1	17/11/2017	Heart Failure	Team meeting	15:30-17:00	1.5
1	16/01/2018	Heart Failure	Team meeting	16:00-17:00	1
	**Total**				**32.5**

Table 2: Documentary Analysis Sources

Document	Initiative			
	MedRev	Wellbeing	Child Allergy	Heart Failure
Meeting minutes	15	13	10	7
Review reports	3	2	3	3
Presentations	2	1	3	3
Webpages	0	0	1	0
Total	**20**	**16**	**16**	**13**

Table 3: Interview and focus group participant list

Participant ID	Initiative	Role/professional role	First round interview	Second round interview	Focus Group
**I1**	Wellbeing	Clinical Lead	Yes	No	No
**I2**	Wellbeing	Clinical Lead	Yes	No	No
**I3**	Wellbeing	Service User	Yes	yes	No
**I4**	Wellbeing	Nurse	Yes	No	No
**I5**	Wellbeing	Service User	Yes	No	No
**I6**	Wellbeing	QI Manager	Yes	yes	No
**I38**	Wellbeing	Doctor	No	yes	No
**I39**	Wellbeing	Project Manager	No	yes	No
**I7**	MedRev	Project Manager (site 1)	Yes	No	Yes
**I8**	MedRev	Patient representative	Yes	No	Yes
**I9**	MedRev	CLAHRC Theme lead	Yes	No	No
**I10**	MedRev	QI Manager	Yes	No	Yes
**I11**	MedRev	Pharmacist, Project manager Site 2	Yes	No	Yes
**I12**	MedRev	Pharmacist, Project manager Site 3	Yes	No	Yes
**I13**	MedRev	Clinical lead	Yes	No	Yes
**I14**	MedRev	Clinical Lead	Yes	No	Yes
**I42**	MedRev	QI Manager	No	No	Yes
**I43**	MedRev	Research Student	No	No	Yes
**I44**	Medrev	Data analyst	No	No	Yes
**I15**	Allergy	Project manager (all sites)	Yes	No	No
**I16**	Allergy	Doctor (both sites)	Yes	No	No
**I17**	Allergy	Embedded researcher	Yes	yes	No
**I18**	Allergy	Clinical Lead (Site B)	Yes	yes	No

**I19**	Allergy	Nurse (Site B)	Yes	Yes	No
**I20**	Allergy	QI Manager	Yes	No	No
**I41**	Allergy	Nurse (Site B)	No	Yes	No
**I37**	Allergy	Nurse (Site A)	No	Yes	No
**I40**	Allergy	Clinical Lead (Site A)	No	Yes	No
**I21**	Heart Failure	QI Manager	Yes	No	No
**I22**	Heart Failure	Service Manager	Yes	Yes	No
**I23**	Heart Failure	Patient Representative	Yes	No	No
**I24**	Heart Failure	Data analyst	Yes	Yes	No
**I25**	Heart Failure	Project Manager	No	Yes	No
**I26**	Heart Failure	Data analyst	No	Yes	No
