# Supplementary material for: Unpacking the ‘process of sustaining’—identifying threats to sustainability and the strategies used to address them: a longitudinal multiple case study
Source: Implement Sci Commun. 2023 Jun 19;4:68. doi: 10.1186/s43058-023-00445-z (PMC10278281; doi:10.1186/s43058-023-00445-z)
Supplement: Supplementary file 3 — Additional file 3. Interview Guides. [file 43058_2023_445_MOESM3_ESM.pdf]

**Supplementary File 3_Interview Guides**

## Interview Guide and Questions

**Introduction:** The aim of this work is to understand the process by which improvement team members influence the sustainability of their projects and to understand participant views on sustainability and long term success. Interviews with team members will be conducted at two time points to gain in-depth understanding of sustainability processes and actions. A purposive sampling strategy will be used to recruit interviewees from improvement teams. We would like to ask you a few questions to aid in our understanding of how improvements are sustained in practice.

Semi-structured interviews will explore perceptions, experiences and actions associated with sustainability. We will not be using your name or any identifiable titles to attribute statements you have made. All data will be anonymised.

**Ethics:** This research protocol and interview questions have been approved by Imperial College HRA IRAS number: 188851.

**Sampling Strategy and Participant Selection**: A purposive sample strategy will be used to select participant. Participants will be selected based on their level of knowledge of the project or intervention and their role in the project. This aims to maximize the diversity of perspectives gained from the interviews. All participants will be CLAHRC NWL stakeholders who 1) *Have been involved in a past project 2) Will be involved in a future project 3) Have been involved in using the NHS III model* and/or the Long Term Success Tool.

**Settings:** CLAHRC NWL Office, Project NHS trust sites, telephone, Skype

**Time:** Approximately 30-45 minutes

**Time frame:** Interviews will be conducted in April-September 2017 and April-September 2018.

**Method:** The interview will be semi-structured, using an interview protocol to guide the interviews. An exploratory approach will be taken to examine any emerging themes and issues therefore revision of the protocol will take place as the research progresses. Based on

participant responses and initial analysis the initial research questions and interview questions will be adapted.

As data are collected and analysed initial coding and interpretations will be discussed with supervisors and select participants. This process will enable interpretations of the data to be shared with the participants, and give the participants the opportunity to discuss and clarify the findings, and contribute further perspectives on the issue under study.

**Analysis:** The analysis will involve the following stages: familiarization, identifying thematic framework, coding, and mapping and interpretation. Transcribed interview notes and recordings will be analysed using Nvivo, a qualitative research software, to identify common challenges and themes across projects and settings.

**Themes covered:**

I. Perceptions of added value of models, tools and frameworks for sustainability
II. Longevity and lasting impacts of initiative improvements
III. Inputs, factors and actions needed to achieve long term success
IV. Risks to Sustainability
V. Use, engagement with and impact of the Long Term Success Tool

## <u>Long Term Success Interview Questions</u>

***First round April-September 2017***

1) Can you please describe you **past experience** with improvement projects?
   I. What did this project aim to achieve and was this accomplished? Why or why not?
   II. Was this project sustained?
   III. Can you briefly describe any ***strategies*** you may have used to try to influence sustainability? (actions taken, models, methods or tools such as the NHS III sustainability model)?
   IV. What did you learn from this experience?

2) Can you please describe your **current improvement project** and its aims?
   I. What is your role in the project?

3) Can you describe any **key improvements/achievements** your project has accomplished to date?
   I. Throughout the project have there been any steps taken to ensure long term success is achieved? If so what were they?

4) A) In your opinion, what has been/will be the **greatest challenge** to the sustainability of your project? Why?

I. Are there any actions that were taken/could be taken to improve this?

B) In your opinion what has been or will be the **greatest facilitator** to the sustainability of your project? Why?

5) Can you describe **your experience** in **using the LTS** approach?
    I. How has it felt compared to previous sustainability strategies you have used?
    II. In what way, if any, has it influenced your project's journey or trajectory?

6) These are the **LTS Tool results** from the project **(show project Spider Diagram and Bar chart**). What are your thoughts on this?
    I. How reflective are the results compared to your experience of being involved in the project?
    II. Has anything been missed?

7) Your reports indicated these challenges /action (**show project challenges/actions list**). Did you or your team follow up on these?
    I. If so, what actions has your team undertaken to address this?
    II. If not, are there any actions that could have been beneficial?

8) Do you believe the improvements your project is making ***will be sustained***?
    I. For how long?
    II. What could improve this?

9) What are the ***next steps*** for this project?

10) Any other comments?

## Follow-up Interview Questions

***April – September 2018***

1. Can you describe what has happened with the heart failure project in the past year?

2. In your opinion has the initiative been sustained?

3. As sustainability can be measured in a number of ways, can you describe based on the descriptions if any of the following have been achieved?

| Sustainability outcomes | y/n, describe |
|---|---|
| I. Benefits or outcomes for patients are continued | |
| II. Continuing the program activities or components of the original intervention | |
| III. Maintaining community-level partnerships or coalitions developed during the funded program | |
| IV. Maintaining new organizational practices, procedures, and policies that were started during program implementation | |
| V. Sustaining attention to the issue or problem | |
| VI. Program diffusion and replication in other sites | |

4. For the previous interview, the aim of your work was to: X.  It included x,y,z.  Has the intervention changed at all from what was initially implemented?

- If so how has it changed and why were these changes necessary?

3. For looking at your previous interviews, many of you identified X, Y, Z as an issue for sustainability. Can you tell me if and how these influenced the project journey in the last year?

- Were there any other challenges the project encountered?

4. Many of your colleagues also identified X, Y, Z and facilitators for sustainability. Can you tell me if and how these influenced the project journey in the last year?

- Where there any other things that helped the project along the way?

6. Some strategies for sustaining were also highlighted in the previous interviews such as X,Y,Z.

- Have these actions taken place?
- Have other actions been taken to aid in the project's sustainability?
- How have they helped?

7. What are the next steps for this project?

8. Any other comments?
